# Superior Ophthalmic Vein Thrombosis Associated With Acute Suppurative Parotitis and Masticator Space Cellulitis: A Case Report and Review of the Literature

**DOI:** 10.7759/cureus.73721

**Published:** 2024-11-15

**Authors:** Hazel Choi, Max Sheng, Michael Chandler

**Affiliations:** 1 Integrative Medical Sciences, Northeast Ohio Medical University, Rootstown, USA; 2 Diagnostic Radiology, University Hospitals Cleveland Medical Center, Cleveland, USA; 3 Pulmonology/Critical Care Medicine, Summa Health, Akron, USA

**Keywords:** critical care, masticator space cellulitis, ophthalmoplegia, superior ophthalmic vein thrombosis, suppurative parotitis

## Abstract

Superior ophthalmic vein thrombosis (SOVT) is a rare phenomenon caused by both septic and aseptic etiologies. Individuals suffering from SOVT commonly present with ophthalmoplegia, proptosis, chemosis, and impaired vision. The superior ophthalmic vein is a valveless vein that drains into the cavernous sinus; for this reason, SOVT can present with dire complications such as cavernous sinus thrombosis in addition to debilitating permanent ophthalmoplegia and permanent vision loss if left untreated. Early accurate diagnosis and treatment of SOVT are of paramount importance in order to improve patient outcomes and to reduce morbidity and mortality. In this paper, the authors present an uncommon case of SOVT associated with acute suppurative parotitis and masticator space cellulitis.

## Introduction

Superior ophthalmic vein thrombosis (SOVT) is a rare disease with a yearly incidence of approximately 3-4 cases per one million [1]. The superior ophthalmic vein (SOV) originates from the orbital fossa and traverses with the superior orbital artery within the intraconal space in order to drain into the cavernous sinus [2]. SOVT can be secondary to septic and aseptic causes. Septic causes include orbital cellulitis, rhinosinusitis, septic cavernous sinus thrombosis (CST), and bacteremia and account for 33% of all cases described in the literature [1,3]. Aseptic causes include hypercoagulable states (homocystinuria, antiphospholipid syndrome, oral contraceptive use, systemic lupus erythematosus), sarcoidosis, malignancies (including hematologic and solid organ malignancies), facial trauma, carotid cavernous fistula, Grave’s disease, high-altitude and Tolosa-Hunt syndrome and account for 67% of all cases described in the literature [3-9]. To our knowledge, there have been no indexed cases of SOVT associated with acute suppurative parotitis and masticator space cellulitis. In this article, we hope to increase awareness surrounding SOVT due to its rareness and to improve patient outcomes.

## Case presentation

The patient is a 67-year-old woman with a past medical history of congestive heart failure and hypertension with a chief complaint of right upper dental pain for one and a half weeks. The patient presented to the emergency department after failing outpatient therapy with doxycycline and dexamethasone. On arrival at the emergency department, the patient experienced pain in the right jaw and face, decreased vision in the right eye, and limited ocular mobility to vertically elevate or abduct the right eye. Initial vital signs showed a mean arterial pressure (MAP) of 54 millimeters mercury (mmHg), a respiratory rate of 16 breaths per minute, a heart rate of 94 beats per minute, and a temperature of 36.2 degrees Celsius. Physical examination was significant for proptosis of the right eye and inability to abduct or elevate the right eye. Left eye movements were normal. The remaining physical examination was unremarkable. Initial remarkable labs on admission include white blood count (WBC) 15.0 x 10^3/µL, hemoglobin 13.4 g/dL, platelets 57 x 10^3/µL, lactic acid 2.4 mmol/L, anion gap 20 mmol/L, bicarbonate 13 mmol/L, creatinine 6.55 mg/dL, procalcitonin 6.88 ng/mL, erythrocyte sedimentation rate (ESR) 70 mm/h and C-reactive protein 12.3 mg/L (Table 1).

**Table 1:** Laboratory values for blood tests at admission.

Laboratory results (units)	Values
White blood cell (/µL)	15.0 x 10^3^
Hemoglobin (g/dL)	13.4
Platelets (/µL)	57.0 x 10^3^
Sodium (mmol/L)	139
Potassium (mmol/L)	3.8
Chloride (mmol/L)	105
Bicarbonate (mmol/L)	13
Anion gap (mmol/L)	20
Creatinine (mg/dL)	6.55
eGFR (mL/min/1.73m*2)	6.5
Albumin (g/dL)	3.3
Total protein (g/dL)	6.5
Aspartate transaminase (U/L)	40
Alanine transaminase (U/L)	27
Alkaline phosphatase (U/L)	224
Bilirubin, Total (mg/dL)	1.3
Lactic Acid (mmol/L)	2.4
C-reactive protein (mg/dL)	12.3
Erythrocyte sedimentation rate (mm/h)	70
Procalcitonin (ng/mL)	6.88

**Figure 1:**
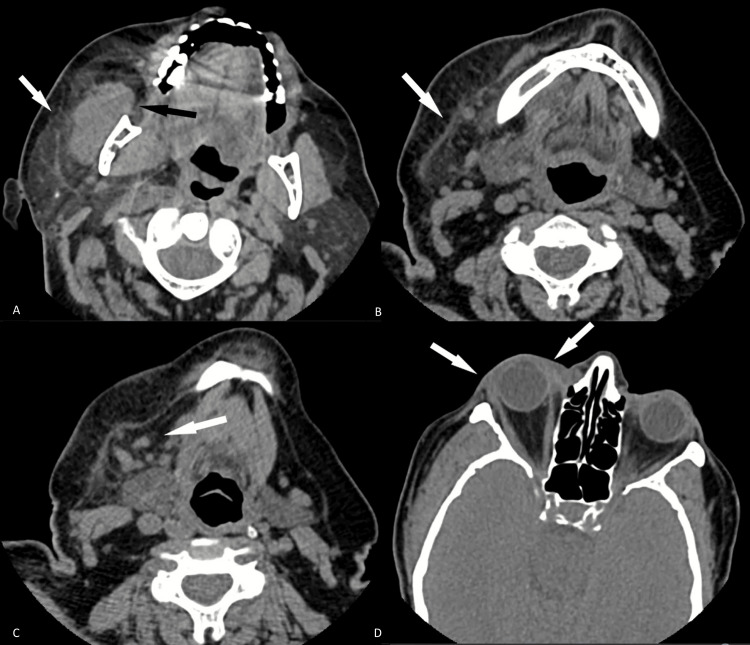
CT images of the maxillofacial skeleton and orbit showing findings of right-sided acute suppurative parotitis and orbital cellulitis. Axial CT of the maxillofacial skeleton shows asymmetric enlargement of the parotid gland with increased adjacent fat stranding consistent with inflammatory changes (white arrow, A) and acute right suppurative parotitis. There is also asymmetric enlargement of the right masseter (black arrow, A) and platysma muscles (white arrow, B) with numerous prominent right level 1B cervical lymph nodes (white arrow, C). Axial CT of the orbit demonstrates increased soft tissue edema and fat stranding within the extraconal palpebrae tissues spanning from the medial to lateral epicanthal folds (white arrows, D) concerning for orbital cellulitis. There was no retrobulbar extension.

Computed tomography (CT) of the head showed no acute intracranial pathology. CT of the maxillofacial skeleton revealed asymmetric enlargement of the right parotid gland with surrounding fat stranding consistent with inflammatory changes raising concern for acute right suppurative parotitis. There was also asymmetric enlargement of the right masseter and platysma muscles and numerous prominent level 1B cervical lymph nodes on the right (Figures 1A-1C). CT of the orbit showed increased soft tissue edema and fat stranding throughout the extraconal palpebrae tissues extending from the medial to lateral epicanthal folds compatible with right orbital cellulitis (Figure 1D). There was no retrobulbar extension. Because of the patient's low MAP with concern for septic shock, the sepsis protocol was initiated, and the patient was fluid resuscitated and started on norepinephrine and broad-spectrum antibiotic coverage with vancomycin and piperacillin/tazobactam. Due to the high concern for CST, the patient was transferred to the medical intensive care unit (MICU) for further medical care.

**Figure 2:**
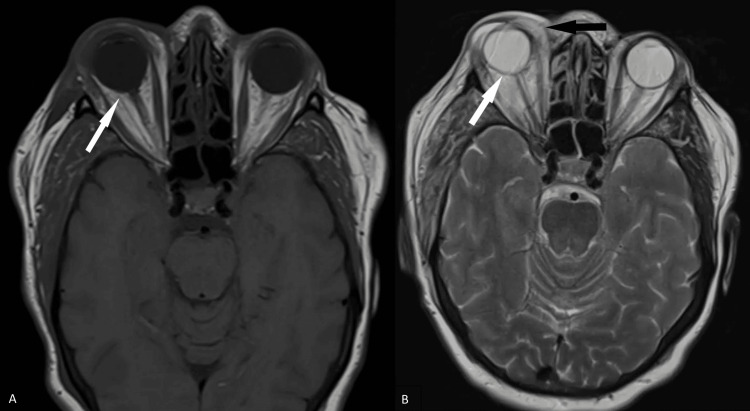
MRI images of the brain showing findings of right orbital cellulitis and increased intraorbital pressure. Axial T1-weighted and T2-weighted sequences demonstrate tenting of the right posterior globe (white arrows, A and B) suggestive of increased right intraorbital pressure. The axial T2-weighted sequence also shows increased T2 signal intensity throughout the extraconal palpebrae soft tissues spanning from the medial to lateral epicanthal folds consistent with right orbital cellulitis (black arrow, B).

**Figure 3:**
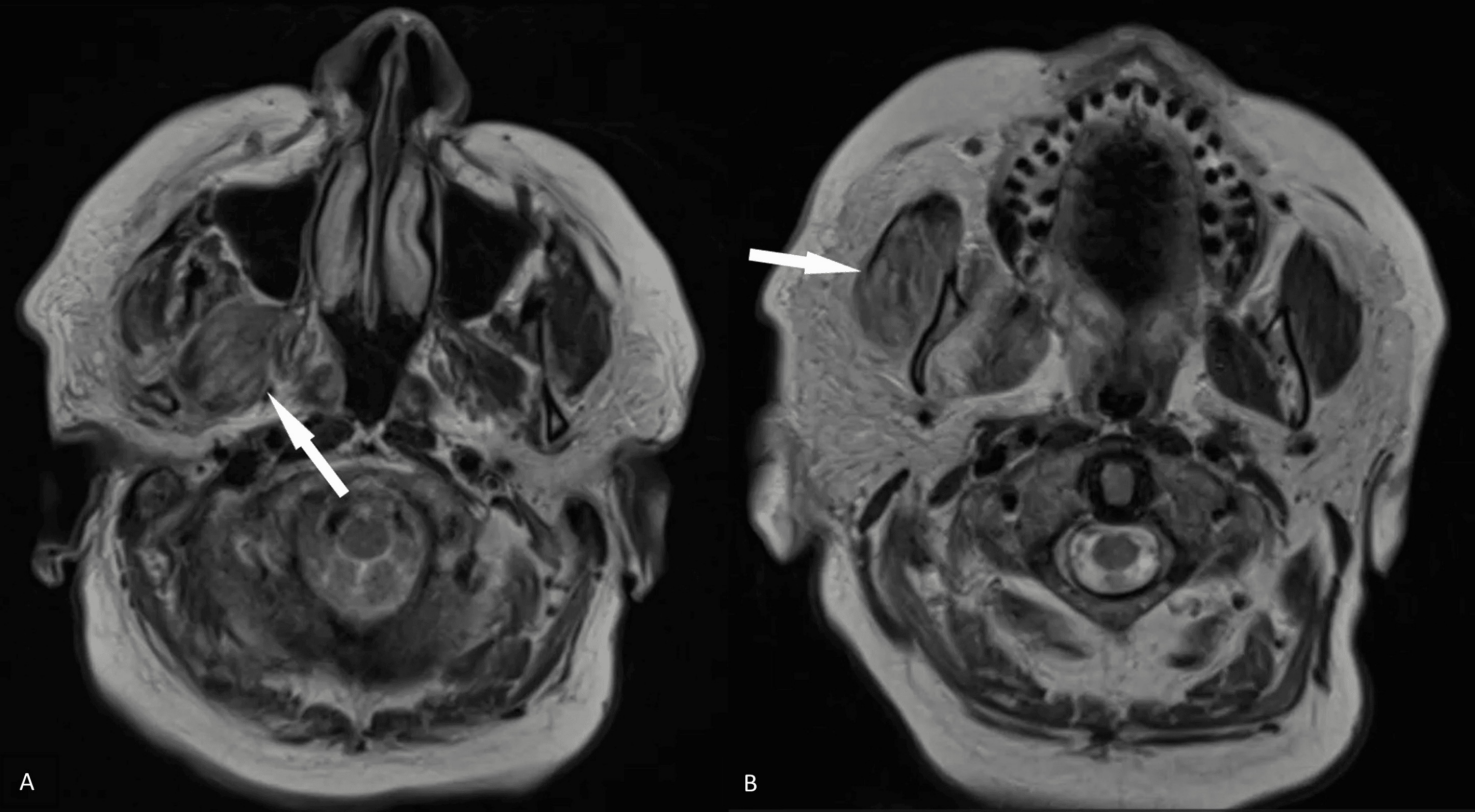
MRI images of the brain showing findings of right masticator space cellulitis. Axial T2-weighted images demonstrate asymmetric enlargement and increased T2 signal intensity within the muscles of the right masticator space, namely the medial pterygoid (white arrow, A) and masseter muscles (white arrow, B) indicative of right masticator space cellulitis.

**Figure 4:**
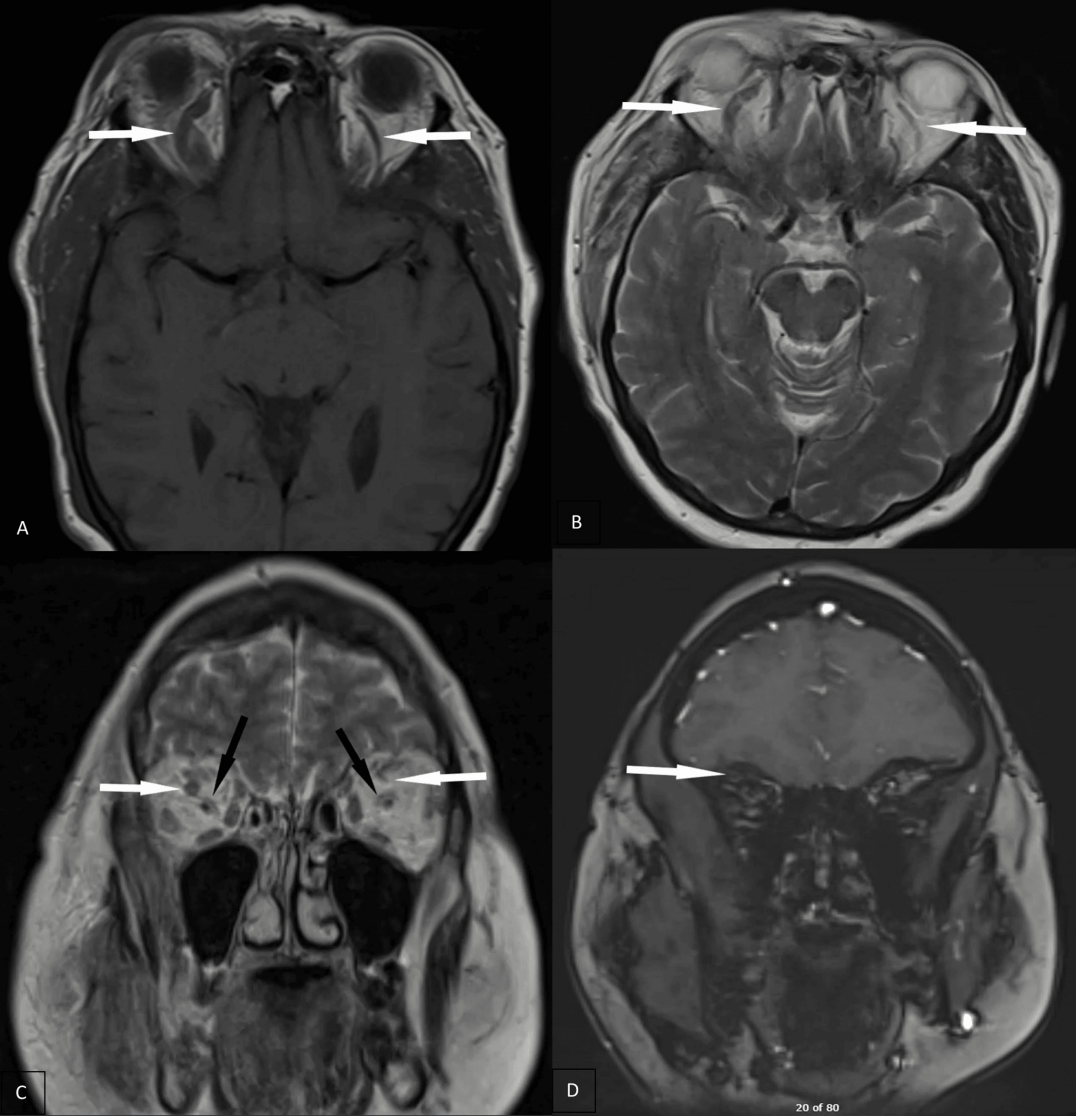
MRI images of the brain showing findings of right superior ophthalmic vein thrombosis. The axial T1-weighted image and axial T2-weighted image demonstrate asymmetric enlargement of the right superior ophthalmic vein (white arrows, A and B). The coronal T2-weighted image shows enlargement of the right superior ophthalmic vein demonstrated in another plane (white arrows, C) with normal appearance of the optic nerve and sheath bilaterally (black arrows, (C). The coronal T1-weighted post-gadolinium image shows a faint intraluminal filling defect within the right superior ophthalmic vein compatible with right superior ophthalmic vein thrombosis (white arrow, D).

Further investigation with magnetic resonance imaging (MRI) of the brain showed tenting of the right posterior globe (Figures 2A, 2B) suggestive of increased right intraorbital pressure in addition to right-sided orbital cellulitis. There was also asymmetric enlargement with increased T2 signal intensity throughout the muscles in the right masticator space, such as the medial pterygoid and masseter muscles, suggestive of right masticator space cellulitis (Figures 3A-3B). Additional enlargement of the right superior ophthalmic vein was present (Figures 4A-4C). Coronal T1 weighted post-gadolinium images revealed an intraluminal filling defect within the right superior ophthalmic vein consistent with right superior ophthalmic vein thrombosis (Figure 4D). During this time frame, the patient also experienced six to seven black and tarry stools which delayed anticoagulation therapy.

After one week, blood cultures returned positive for Streptococcus intermedius bacteremia. Infectious disease was consulted and resolved that streptococcus bacteremia and septic shock initially started as right-sided acute suppurative parotitis progressing to right masticator space cellulitis and ascending superiorly precipitating in right orbital cellulitis and right superior ophthalmic vein thrombosis. Due to overwhelming septicemia, the patient developed acute respiratory distress syndrome requiring intubation. Additional labs revealed Clostridium Difficile (C Diff.) glutamate dehydrogenase positivity and 100,000 (CFU/mL) of Klebsiella pneumoniae on urine culture concerning for superimposed C Diff. colitis and urinary tract infection. Cerebrospinal fluid studies and additional microbiology cultures were negative. Transthoracic echocardiography showed normal left ventricular systolic function without valvular vegetations. Ophthalmology was consulted and the patient was treated with dexamethasone, vancomycin, ceftriaxone and metronidazole combination therapy. The patient was hospitalized in the MICU for approximately one month and improved significantly towards the end of the admission. 

**Figure 5:**
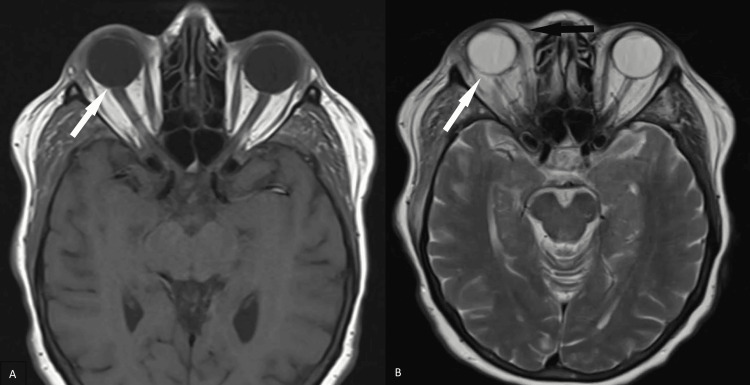
MRI images of the brain showing findings of resolved increased right intraorbital pressure and right orbital cellulitis. Axial T1-weighted and T2-weighted sequences demonstrate resolution of right posterior globe tenting (white arrows, A and B) suggestive of right intraorbital pressure normalization. The axial T2-weighted sequence also demonstrates normalization of T2 signal intensity throughout the extraconal palpebrae soft tissues overlying the epicanthal folds indicative of right orbital cellulitis resolution (black arrow, B).

**Figure 6:**
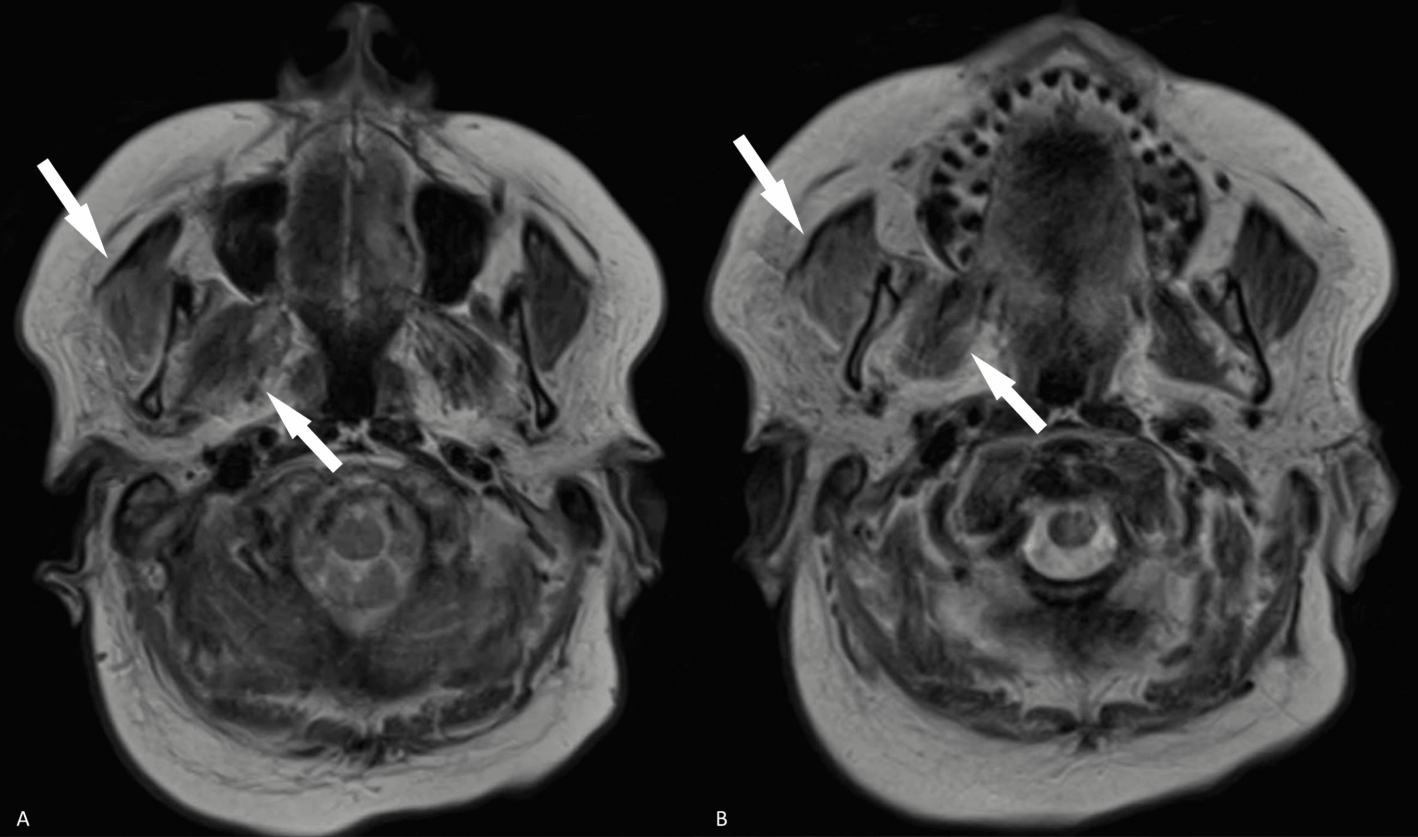
MRI images of the brain showing findings of resolved right masticator space cellulitis. Axial T2-weighted images demonstrate resolution of abnormal T2 signal intensity within the muscles of the right masticator space (white arrows, A and B) compatible with resolved right masticator space cellulitis.

**Figure 7:**
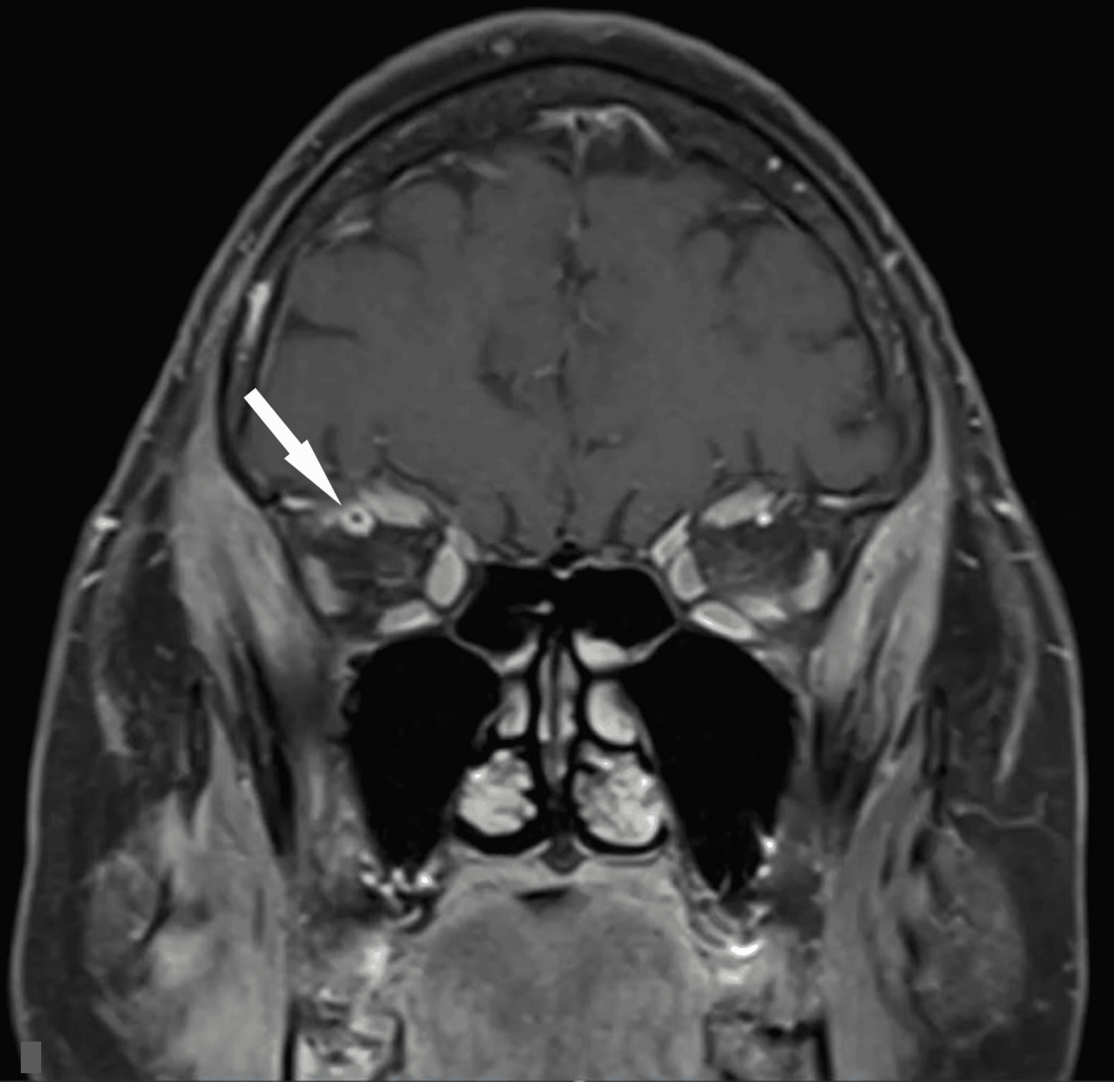
MRI images of the brain showing findings of persistent right superior ophthalmic vein thrombosis. The coronal T1-weighted post-gadolinium image shows a persistent intraluminal filling defect within the right superior ophthalmic vein compatible with persistent right superior ophthalmic vein thrombosis (white arrow, A).

Near the time of discharge, the patient's leukocytosis and anion gap have resolved with near normalization of the remaining laboratory values (Table 2). MRI images of the brain showed resolved elevated right intraorbital pressure and right orbital cellulitis (Figures 5A, 5B) as well as right masticator space cellulitis (Figures 6A, 6B). However, thrombosis within the right superior ophthalmic vein persisted at the time of discharge (Figure 7). The patient was discharged to a long-term acute care facility to complete one-week course of oral vancomycin, four-to-six-week course of ceftriaxone, and three-month course of apixaban.

**Table 2:** Laboratory values for blood tests at discharge.

Laboratory results (units)	Values
White blood cell (/µL)	9.5 x 10^3^
Hemoglobin (g/dL)	7.9
Platelets (/µL)	391.0 x 10^3^
Sodium (mmol/L)	133
Potassium (mmol/L)	3.6
Chloride (mmol/L)	102
Bicarbonate (mmol/L)	29
Anion gap (mmol/L)	2
Creatinine (mg/dL)	0.59
Albumin (g/dL)	2.5
Total protein (g/dL)	5.6
Aspartate transaminase (U/L)	47
Alanine transaminase (U/L)	18
Alkaline phosphatase (U/L)	61
Bilirubin, Total (mg/dL)	0.3

## Discussion

The SOV is formed by the union of the angular, supraorbital, and supratrochlear veins [10]. The SOV courses in between the superior rectus muscle, optic nerve, and ophthalmic artery in the intraconal space and exits via the superior orbital fissure to the annulus of Zinn between the frontal (V1 branch of the trigeminal nerve) and trochlear nerves [10]. The SOV provides venous drainage to the globe, upper eyelid, extraocular muscles, and lacrimal gland [10]. The mean diameter of the SOV is around 2 mm with normal sizes ranging from 1 to 2.9 mm [11]. The SOV remains a clinically relevant structure because it is a valveless vein and drains directly into the cavernous sinus [4], as such, any infection or thrombus in the SOV has a direct communicating tract with the cavernous sinus. 

SOVT is a rare entity and is infrequently reported in the literature. Its cause has been separated into septic and aseptic dichotomies with aseptic causes being more frequently described in literature. The most common septic cause for SOVT is orbital cellulitis [1]. The pathophysiology surrounding SOVT development is poorly understood; however, it has been attributed to anatomic or iatrogenic alterations to venous blood flow in the SOV and disruption of Virchow’s triad [6,7,12]. SOVT can be unilateral or bilateral. Patients with SOVT present with orbital swelling, limited ocular mobility, chemosis, eyelid edema, proptosis, and impaired visual acuity [7]. Diagnosis of SOVT is reliant on clinical reasoning and imaging modality, specifically CT head, MRI brain, and MRV head [2]. 

Parotitis refers to an inflammatory process involving the parotid glands either unilaterally or bilaterally [13]. It can be due to an obstructing stone or occur on its own. Acute unilateral parotid swelling is more commonly seen and is likely due to infectious etiologies. Bilateral parotitis is more commonly due to systemic causes such as autoimmune disease (chronic recurrent parotitis, Sjogren disease), granulomatous disease (sarcoidosis, granulomatosis with polyangiitis), Kimura’s disease, and neoplastic processes [13]. While acute infectious parotitis may occur bilaterally, it is much less common. The most common cause of acute unilateral parotitis is acute suppurative parotitis. Acute suppurative parotitis is an ascending ductal infection that can be caused by an obstructing stone in Stenson’s duct after colonization of the oral cavity by microorganisms or through a hematogenous route from another source [14]. It is most commonly seen in debilitated and dehydrated patients with poor oral hygiene [13]. Staphylococcus aureus is the most common causative agent, however, both gram-positive and gram-negative organisms have been previously described [14]. If left untreated, acute suppurative parotitis may lead to parotid abscess formation, fistulization, mediastinitis, septicemia, and meningitis [14].

Treatment of SOVT is based on prior case reports and physician judgment because no current large-scale studies have been conducted due to this condition’s rarity [15]. Current treatment options include anticoagulation, antibiotics, or surgery without a definitive consensus. Early management is paramount to avoid the major complications which include permanent vision impairment, permanent ophthalmoplegia, CST, and Lemierre’s syndrome [2,6,16]. In prior SOVT case reports with associated orbital inflammation, treatment with glucocorticoids with or without anticoagulation therapy has been used successfully to minimize or prevent vision loss and ophthalmoplegia [17]. The use of glucocorticoids, commonly dexamethasone, may reduce orbital edema which relieves pressure on the orbital structures and reduce structural damage during dissolvement of thrombi [17,18]. However, the prothrombotic effects of glucocorticoids must also be taken into consideration as there is more than a 3-fold increase in risk for venous thrombus formation in patients being treated with glucocorticoids [19]. In previous case reports, glucocorticoid usage in SOVT patients led 10 out of 15 patients to regain good vision suggesting that glucocorticoids played an overall beneficial role in the treatment of SOVT [17]. The role of anticoagulation in both SOVT and CST has remained controversial but appears to reduce morbidity if initiated early [15]. Multiple case reports have suggested the necessity of anticoagulation therapy for aseptic SOVT to prevent progression of CST. However, in cases of septic SOVT, the use of anticoagulation may also prove useful in ways of clot stabilization and canalization of thrombus which improves penetration of antibiotics [15]. Specifically, prior case reports described successes with unfractionated heparin (UFH) and low-molecular-weight heparin (enoxaparin) in treating SOVT [18]. Based on this review of currently published literature, management of SOVT is best achieved with a combination regimen of appropriate antibiotics, anticoagulants, and steroids [20] which showed the greatest improvement in patient outcomes and complication reduction. 

## Conclusions

SOVT is a rare manifestation defined as thrombus formation within the valveless SOV and is of critical importance due to its direct communication with the cavernous sinus. There have been numerous case reports of both septic and aseptic causes of SOVT; however, our case report describes an uncommon manifestation of SOVT associated with acute suppurative parotitis and masticator space cellulitis and its sequelae of complications that follow. Based on current published literature, a combined regimen of anticoagulants (UFH or enoxaparin), dexamethasone, and appropriate antibiotics may prove most beneficial in mitigating lasting complications from SOVT including permanent vision loss, permanent ophthalmoplegia, CST, and Lemierre’s syndrome. Further education and awareness around SOVT are of foremost importance as they will lead to improved patient outcomes surrounding this rare condition.
